# VETERAN MORTALITY DISADVANTAGE AMONG RURAL, SUBURBAN, AND URBAN RESIDENTS

**DOI:** 10.1093/geroni/igz038.1426

**Published:** 2019-11-08

**Authors:** Janet M Wilmoth, Scott D Landes, Andrew S London

**Affiliations:** Syracuse University, Syracuse, New York, United States

## Abstract

Although veterans tend to have higher mortality rates than non-veterans, recent research suggests there is substantial heterogeneity in veteran mortality on the basis of various characteristics such as race, period of service, type of health insurance coverage, and service-connected disability status. This analysis extends the extant literature by using the 1978-2014 General Social Survey linked to the National Death Index (GSS-NDI) to examine veteran status differences in mortality by geographic location. We estimate a series of Cox regression models predicting death for male veterans and nonveterans, controlling for age, race/ethnicity, education, income, and work status. Separate models are presented for rural, suburban, and urban residents. All models are weighted and include robust standard errors. The results indicate that rural veterans have higher mortality risk than rural non-veterans, particularly among older adults. There are no significant differences in mortality risk between veteran and non-veterans living in suburban and urban areas.

